# Unveiling the Chasm in Japanese robot-assisted surgery diffusion: the interplay of surgical volume and intra-specialty spillover

**DOI:** 10.1007/s11701-026-04049-2

**Published:** 2026-10-12

**Authors:** Yusuke Natsume, Shingo Kano

**Affiliations:** Bio-Innovation Policy Unit, Department of Computational Biology and Medical Sciences, Graduate School of Frontier Sciences, The University of Tokyo, 5-1-5 Kashiwanoha, Chiba 277-8562 Kashiwa City, Japan

**Keywords:** Robot-assisted surgery, Diffusion of innovation, NDB Open Data, Cross-specialty analysis, Chasm, Spillover effect

## Abstract

To examine structural factors associated with the diffusion of robot-assisted surgery (RAS) in Japan, this study examined the associations of annual surgical volume, defined as the sum of open, conventional laparoscopic, and RAS cases, and prior intra-specialty experience with adoption speed. Using the 1st to 10th editions of the National Database of Health Insurance Claims and Specific Health Checkups of Japan (NDB) Open Data across fiscal years 2014–2023, diffusion trajectories were visualized for 21 procedures approved for insurance coverage by 2020 through bivariate plots of annual surgical volume versus RAS replacement rates. RAS diffusion exhibited non-linear dynamics associated with annual procedure volume. In niche markets with < 6,000 annual cases (*n* = 9), six procedures demonstrated high growth in RAS replacement rate. Conversely, among 12 procedures with ≥ 6,000 annual cases, four did not cross the 16.0% chasm threshold through FY2023, and three required 6 years to surpass it. These patterns were thought to reflect challenges in the transition from specialized centers to general hospitals. Furthermore, a trend consistent with “intra-specialty spillover” was observed, wherein procedures approved in FY2020 achieved higher initial adoption than those in FY2018; although inseparable from the overall expansion of RAS since 2018, this suggests that accumulated infrastructure and expertise may facilitate the uptake of subsequent techniques. These findings suggest that RAS diffusion is associated with annual procedure volume, organizational readiness, and specialty‑specific infrastructure alongside technical factors. Future implementation strategies may benefit from infrastructure sharing within and across specialties and resource optimization to bridge the observed diffusion chasm.

**Supplementary Information:**

The online version contains supplementary material available at https://doi.org/10.1007/s11701-026-04049-2.

## Introduction

### Global emergence and evolution of robot-assisted surgery

Robot-assisted surgery (RAS) has undergone remarkable evolution since the early prototypes, the PUMA 560 in 1985 and the AESOP in 1994 [1, 2]. These early systems initially served auxiliary roles, such as stabilizing endoscopes or guiding biopsies, but the introduction of the da Vinci Surgical System in 1999 marked a decisive technological shift. By combining high-definition 3D visualization with articulated instruments (EndoWrist) capable of surpassing the dexterity of the human wrist, the master–slave architecture transformed RAS from a supportive tool into a true extension of the surgeon’s hands [2, 3]. Regulatory milestones soon followed, including United States Food and Drug Administration approval for general surgery in 2000 and radical prostatectomy in 2001, catalyzing global adoption [2, 3]. As of late 2022, more than 7,000 units were in operation worldwide [3, 4], with Japan emerging as the second‑largest market after the United States, deploying over 570 units by 2023 [4]. Despite this widespread implementation, the structural mechanisms through which RAS replaces conventional techniques and stabilizes within clinical practice remain poorly understood [2].

### Theoretical framework and gaps in international evidence

Within classical diffusion-of-innovations theory, transitioning from early adopters to the early majority, defined by the cumulative 16.0% threshold, represents a critical juncture where the primary driver of adoption shifts from specialized visionaries seeking technological innovation to pragmatic, mainstream institutions demanding clinical reliability, cost-effectiveness, and standardized training [5, 6]. Consequently, crossing this 16.0% boundary often encounters a structural barrier known as the “chasm” [3, 7]. Internationally, urology has been a primary driver of RAS adoption accounting for nearly one‑third of influential RAS publications [1]. In the United States, robot‑assisted radical prostatectomy reached an 85% replacement rate by 2013 [8], indicating diffusion into even the laggard segment and establishing RAS as the de facto standard of care for this procedure [3]. However, despite these well‑documented successes, two primary research gaps remain.

First, many analyses have remained “siloed” within individual specialties, overlooking how infrastructure, training, and institutional experience in one field may accelerate adoption in others, a phenomenon often described as “multispecialty expansion” [9, 10]. Second, comprehensive data coverage is lacking. The Nationwide Inpatient Sample (NIS) in the United States captures only around 20% of annual discharges, while National Health Service (NHS) datasets in the United Kingdom exclude private‑sector activity, limiting the generalizability of diffusion estimates [8, 9, 11]. As a result, macro‑level structural determinants of RAS diffusion remain poorly quantified.

### RAS implementation in japan: opportunities and unresolved questions

Japan presents a unique environment for studying diffusion dynamics. Since the first insurance coverage for RAS in 2012, adoption has expanded rapidly through institution‑led implementation [2, 12]. Urology has spearheaded this movement, with robot‑assisted radical prostatectomy reaching saturation and becoming the standard of care [2–4]. In contrast, diffusion in other specialties, such as gynecology, has progressed more slowly despite expanded coverage in 2018 [13]. While domestic studies cite financial viability and limited training opportunities as barriers, the relationship between these factors and the “annual procedural volume” of each technique remains unclear [4, 13].

### Advantages of NDB open data for cross-specialty analysis

The National Database of Health Insurance Claims and Specific Health Checkups of Japan (NDB) Open Data provides a valuable opportunity to address these gaps. Covering more than 95% of Japanese medical claims, it enables comprehensive, bias‑minimized analysis of surgical practice patterns at a nationwide scale [14]. By utilizing NDB Open Data to determine the annual surgical volume for each procedure, it becomes possible for the first time to quantitatively evaluate the diffusion trajectory of RAS across multiple specialties [14].

### Objectives

This study aims to clarify the current status of RAS diffusion by analyzing cross-specialty adoption using nationwide NDB Open Data [14]. Specifically, we seek to:

1. evaluate how annual total surgical volume relates to the speed and trajectory of RAS adoption in procedures that have not yet reached saturation; and.
2. examine an intra‑specialty spillover pattern, whereby experience accumulated from preceding procedures is associated with faster adoption of subsequent ones.

By addressing these objectives, the study contributes to a more comprehensive theory of replacement‑based diffusion and provides practical implications to inform educational resource allocation, infrastructure planning, and health‑policy design.

## Methods

### Dataset and procedure selection

This study employed descriptive statistics on national-level administrative claims data to evaluate the diffusion dynamics of RAS across multiple specialties in Japan. The objective was to examine cross-specialty patterns of RAS diffusion as a technological platform, without directly evaluating specialty‑specific clinical factors [2].

#### Data source and study period

The analysis was based on the NDB Open Data, a comprehensive repository covering more than 95% of all medical claims generated under Japan’s universal health insurance system [14, 15]. The study period spanned 10 fiscal years, from FY2014 to FY2023 (April 2014 to March 2024), using the 1st to 10th editions of the NDB Open Data published annually.

From all RAS procedures approved during this period, we identified 21 major procedures that received insurance coverage by FY2020 (Table 1) [2].

**Table 1:** List of robot-assisted surgical procedures included in the study by year of National Health Insurance (NHI) coverage

Clinical Specialty / Year of NHI coverage	2012	2016	2018	2020
Urology	・Radical prostatectomy	・Radical nephrectomy and nephroureterectomy	・Radical cystectomy	・Pyeloplasty
Thoracic surgery	—	—	・Lobectomy (Malignant tumor surgery) ・Resection of malignant mediastinal tumor ・Resection of benign mediastinal tumor	・Segmentectomy (Malignant tumor surgery) ・Extended thymectomy
Gastrointestinal surgery	—	—	・Esophagectomy ・Gastrectomy (Simple resection / Malignant tumor surgery) ・Proximal gastrectomy ・Total gastrectomy ・Rectal resection and amputation	・Mediastinoscopic esophagectomy
Gynecology	—	—	・Vaginal hysterectomy ・Radical hysterectomy (limited to endometrial cancer)	・Sacrocolpopexy
Cardiac surgery	—	—	・Valvuloplasty	—
Hepatobiliary-pancreatic surgery	—	—	—	・Pancreaticoduodenectomy ・Distal pancreatectomy

#### Identification and classification of surgical procedures

Surgical procedures were identified using the nine‑digit “Medical Practice Codes” defined in the NDB, mapped to the broader “K-code” classifications of the Japanese medical fee system [14]. For each procedure, we systematically categorized and aggregated multiple practice codes representing open, laparoscopic, and RAS according to their specific clinical content. This approach ensured an accurate classification of open, laparoscopic, and robotic performed for the same clinical indication. The complete mapping of practice codes and the standardization logic for all 21 procedures are documented in Online Resource 1 to ensure methodological transparency.

Two procedures, namely thoracoscopic resection of benign mediastinal tumor and mediastinoscopic esophagectomy, lack independent “open surgery” codes within the NHI system. For these procedures, the study population was defined as the combined total of laparoscopic/thoracoscopic and RAS cases.

#### Classification of interdisciplinary procedures

“Sacrocolpopexy,” an interdisciplinary procedure, was classified under gynecology for this analysis. This approach aligns with international methodology used in the literature, drawing on the United States Nationwide Inpatient Sample (NIS) as well as Japanese nationwide surveys on robotic surgery in obstetrics and gynecology [10, 13]. Although Japan maintains a formal governance structure through professional societies such as the Japan Society for Endoscopic Surgery (JSES) to oversee surgical quality, this classification ensures international comparability and statistical consistency [2, 10, 13].

### Evaluation metrics for RAS diffusion

#### Metrics for technological maturity (RAS Replacement Rate)

To quantify RAS adoption, two metrics were defined: total surgical volume and RAS replacement rate. Total volume represented the annual sum of open, endoscopic (laparoscopic/thoracoscopic), and RAS procedures. The RAS replacement rate was calculated as the percentage of RAS cases within the total volume. Under the NDB disclosure policy, annual counts fewer than 10 were treated as zero [14].

#### Bivariate analysis of adoption trajectories

Diffusion trajectories were evaluated using bivariate plots with RAS replacement rate on the horizontal axis and annual total surgical volume on the vertical axis. To highlight specialty-specific trends, procedures were categorized into five clinical groups, namely urology, thoracic surgery, gastrointestinal surgery, gynecology, and a fifth group comprising cardiac and hepatobiliary-pancreatic surgery, and presented as a multi-panel figure. Annual data were plotted from the year of NHI approval through FY2023. For robot-assisted radical prostatectomy, analysis began in FY2016, the earliest year with consistently identifiable data [14].

Iso-volume reference lines at 5,000, 8,000, and 10,000 annual cases were superimposed to visualize adoption progress relative to overall procedural scale across clinical specialties.

In this study, the “Chasm”, defined as the theoretical boundary between early adopters and the early majority, was operationalized at a cumulative replacement rate threshold of 16.0% (combining 2.5% Innovators and 13.5% Early Adopters) based on classical diffusion theory. Rather than treating 16.0% as an empirically optimized statistical cutoff, this baseline was demarcated on the bivariate plots using a red dashed line as a theoretical reference threshold to visually evaluate adoption progress across specialties.

### Assessment of intra-specialty spillover

To assess the intra-specialty spillover pattern, in which pre-existing expertise and infrastructure within a clinical department may facilitate the adoption of subsequent techniques, we analyzed procedures that received NHI coverage in FY2020 across urology, thoracic surgery, gastrointestinal surgery, and gynecology. These procedures were categorized into two groups: the “Preceding Group,” which included specialties with other robot-assisted procedures approved in FY2018, and the “Non-preceding Group.” By comparing the RAS replacement rates during the first year of insurance coverage for each procedure, we examined whether the accumulation of existing platforms and human resources was associated with faster adoption of later-approved procedures [9].

## Results

### Expansion of indications and insurance coverage (2012–2020)

The 21 procedures analyzed in this study, categorized by clinical specialty and insurance coverage year, are detailed in Table 1. The NHI coverage for RAS in Japan began in FY2012 with radical prostatectomy, followed by partial nephrectomy in FY2016, establishing urology as the early driver of implementation. A major medical fee revision in FY2018 simultaneously approved 12 procedures across thoracic surgery, gastrointestinal surgery, gynecology, and cardiac surgery. By the end of FY2020, following an additional seven approvals, the distribution of the 21 insured procedures was as follows: gastrointestinal (6), thoracic (5), urology (4), gynecology (3), hepatobiliary-pancreatic (2), and cardiac surgery (1). These data show that RAS implementation in Japan has expanded from specific niches to the broader surgical field through phased regulatory integration.

### Bivariate adoption trajectories

**Fig. 1:**
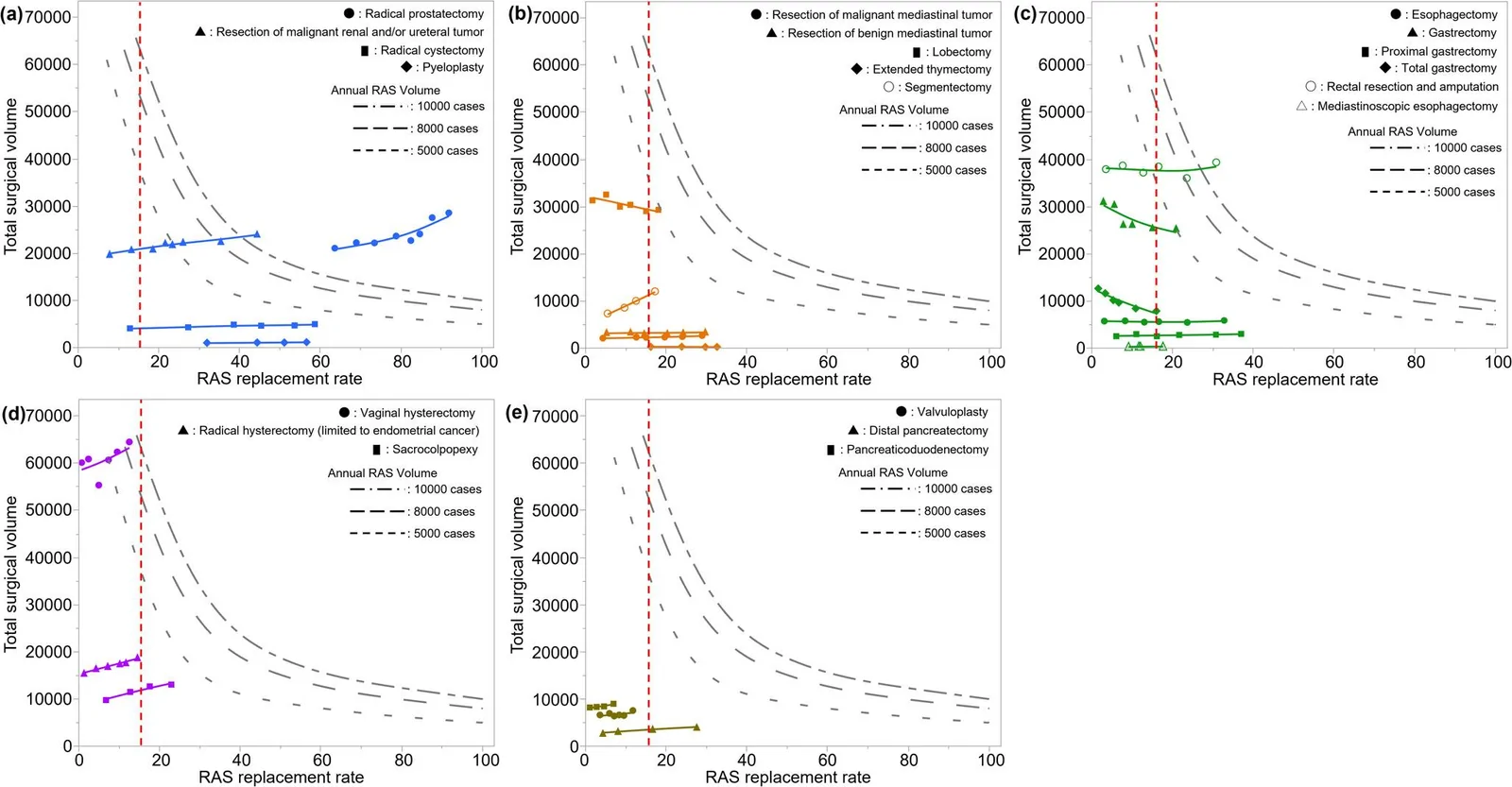
Specialty-specific diffusion trajectories of robot-assisted surgery relative to the 16.0% replacement threshold in Japan. Trajectories represent the relationship between the robot-assisted surgery (RAS) replacement rate (X-axis, %) and the total annual surgical volume (Y-axis, cases/year; sum of open, conventional endoscopic, and RAS procedures) for 21 major procedures from FY2014 to FY2023. (**a**) Urology, (**b**) Thoracic surgery, (**c**) Gastrointestinal surgery, (**d**) Gynecology, and (**e**) Others (cardiothoracic surgery and hepatobiliary-pancreatic surgery). Individual surgical procedures within each panel are distinguished by markers positioned adjacent to the procedure names, utilized in the order of ●, ▲, ▮, ◆, 〇, and △. Reference curves representing annual RAS volumes of 5,000, 8,000, and 10,000 cases are superimposed. The red dashed line marks the 16.0% replacement threshold, used as a theoretical reference for the Chasm derived from classical diffusion theory

The bivariate plots (Fig. 1) illustrate the diffusion dynamics of the 21 procedures through a multi-panel format divided into five clinical specialty categories (urology, thoracic surgery, gastrointestinal surgery, gynecology, and others), with the horizontal axis representing the RAS replacement rate and the vertical axis representing the annual total surgical volume. Radical prostatectomy, the primary benchmark for RAS in Japan, reached a replacement rate of 91.9% by FY2023, demonstrating a growth curve distinct from all other procedures. The iso-volume lines at 5,000, 8,000, and 10,000 annual cases integrated into each panel allow readers to easily estimate the absolute annual volume of RAS procedures, thereby enabling a cross-specialty evaluation of adoption progress.

#### Progress of annual procedure volume in major specialties

Rectal resection and amputation (gastrointestinal surgery) demonstrated substantial expansion, with its trajectory crossing the 10,000-case reference curve as adoption increased. This movement indicates that RAS has become a major implementation pathway in this high-volume domain, echoing the earlier trajectory observed in urology.

#### Rapid replacement in small-scale markets

Procedures with annual volumes below 6,000 cases showed relatively rapid adoption. Average annual growth in RAS replacement rate from first‑year approval to FY2023 was ≥ 5.0% for six procedures: malignant bladder tumor surgery, malignant esophageal tumor surgery, proximal gastrectomy, pyeloplasty, distal pancreatectomy, and thoracoscopic benign mediastinal tumor surgery. All six procedures belong to market segments with < 6,000 annual cases, indicating that RAS adoption was more rapid in these niche segments.

#### Diffusion trajectories in larger-volume procedural markets (≥ 6,000 Cases/Year)

In procedural segments with an annual surgical volume of 6,000 cases or more, adoption dynamics differed markedly from the rapid replacement observed in niche (< 6,000 cases) markets. When evaluated against the 16.0% theoretical reference threshold (demarcated by the red dashed line in Fig. 1), distinct adoption patterns emerged.

Specifically, four procedures, namely vaginal hysterectomy, radical hysterectomy (limited to endometrial cancer), valvuloplasty, and pancreaticoduodenectomy, did not cross the 16.0% threshold through FY2023, remaining below this theoretical reference point.

Conversely, three procedures, namely lobectomy, gastrectomy, and total gastrectomy, crossed the 16.0% baseline in FY2023, taking 6 years after their initial NHI coverage in 2018 to surpass this theoretical threshold.

### Intra‑specialty spillover patterns

To compare adoption patterns by prior specialty‑specific experience, first‑year replacement rates for procedures approved in FY2018 were compared with those approved in FY2020. Across urology, thoracic surgery, gastrointestinal surgery, and gynecology, FY2020 procedures consistently showed higher initial adoption rates.

Examples include:

- Urology: pyeloplasty (31.9% in 2020) vs. radical cystectomy (12.8% in 2018).
- Thoracic surgery: extended thymectomy (16.2%) and lung segmentectomy (5.5%) vs. lung lobectomy (1.7%) and mediastinal tumor resections (4.3% and 5.2%).
- Gastrointestinal surgery: mediastinoscopic esophagectomy (9.2%) vs. gastrectomy (2.9%), total gastrectomy (1.6%), esophagectomy (3.1%), and rectal resection (3.5%).
- Gynecology: sacrocolpopexy (6.7%) vs. malignant uterine tumor resection (1.3%) and hysterectomy (0.7%).

Across all four specialties, procedures approved in FY2020 began their diffusion trajectories at higher initial replacement rates than those approved in FY2018.

## Discussion

### Summary of key findings

This study utilized nationwide NDB Open Data to visualize cross-specialty diffusion patterns for 21 RAS procedures in Japan, revealing three key empirical observations. First, in low-volume procedures with < 6,000 annual cases, rapid replacement occurred, with six procedures demonstrating a mean annual growth in replacement rate exceeding 5.0%. Second, in larger-volume procedural markets (≥ 6,000 annual cases), adoption dynamics differed markedly when evaluated against the 16.0% theoretical reference threshold (the “chasm”): four procedures (vaginal hysterectomy, radical hysterectomy (limited to endometrial cancer), valvuloplasty, and pancreaticoduodenectomy) failed to surpass this 16.0% baseline through FY2023, while three procedures (lobectomy, gastrectomy, and total gastrectomy) required 6 years following initial insurance coverage in 2018 to cross this threshold. Third, we observed a pattern consistent with “intra-specialty spillover,” in which procedures newly approved in FY2020 consistently exhibited higher first-year replacement rates compared to those approved in FY2018 within the same clinical specialties.

### Total annual surgical volume and the adoption paradox: rapid replacement within specialized domains

A striking observation was that RAS replacement progressed most rapidly in niche markets with < 6,000 annual cases. While Rogers’ theory often assumes that larger volumes lead to broader acceptance and faster diffusion [7], this analysis observed a “paradox of narrowness,” in which limited market size correlated with higher adoption rates. This may reflect three factors. First, procedures with smaller annual volumes in Japan are structurally concentrated in a limited number of designated cancer care hospitals and specialized centers under national healthcare policies [16, 17]. Second, highly specialized or rare procedures are often centralized in urban hub hospitals where resident proctors and robotic platforms are already concentrated [2, 4]. When these few specialized institutions transition to RAS, the statistical replacement rate for the entire national market can rise rapidly. Third, the professional community for these procedures is compact, enabling rapid consensus-building and knowledge exchange through academic activities. Social network theory suggests that systems with high homophily and short network distances achieve faster information synchronization [2, 18].

As a result, in niche markets such as pyeloplasty and malignant bladder tumor surgery, the adoption may have progressed more rapidly than in larger-scale markets.

### The Chasm in larger-volume procedures

In diffusion theory, a critical gap known as the “chasm” (defined at a 16.0% replacement rate threshold) exists during the transition from early adopters to the early majority [6, 7]. In our cross-specialty analysis, among procedures with a larger annual volume of ≥ 6,000 cases, we identified procedures that either failed to cross this 16.0% chasm threshold or required up to 6 years to surpass it. Network science models suggest that as the target of adoption expands and the structural distance within the system increases, the time required for information synchronization increases [18]. A similar network dynamic may be hindering the diffusion of these larger-volume procedures. Possible contributing factors include the following two mechanisms.

The first is economic feasibility. In Japan, the break-even point for a surgical robot is estimated to require 150–300 procedures per year at a single institution [12]. However, under the NHI scheme, reimbursement rates for robotic procedures are tied to those of conventional laparoscopic surgeries [2, 12]. Consequently, hospitals must maintain high utilization rates to amortize the substantial capital and maintenance costs, which may be difficult for individual general hospitals to sustain within a single medium-volume indication, potentially creating an economic barrier. This structural mismatch is consistent with survey findings showing that 94.1% of obstetricians and gynecologists identified healthcare economics as the primary barrier [13].

The second is the availability of trained personnel. Proctor-certified physicians remain concentrated in large academic centers, slowing the chain of technical acquisition in general hospitals [4, 13]. Addressing this chasm may require shifting from siloed operations toward multidisciplinary “shared robotic infrastructure” to systematically maximize hospital-level utilization density.

Furthermore, for the four procedures that did not cross the 16.0% threshold through FY2023, specialty-specific clinical and structural hurdles may have compounded these systemic bottlenecks:

1. Gynecologic Procedures (Vaginal Hysterectomy and Radical Hysterectomy (limited to endometrial cancer)): Diffusion may be influenced by case dispersion across regional community hospitals [13, 15], coupled with the established proficiency and preference for conventional laparoscopic techniques among experienced gynecologists [15, 19].
2. Valvuloplasty: Limited adoption may reflect the established role of conventional median sternotomy as the standard approach, a clinical pattern mirrored internationally, where conventional open sternotomy continues to account for over 70% of mitral valve procedures even in the United States [20, 21].

Pancreaticoduodenectomy: Persistent reliance on open surgery, a trend also mirrored internationally where over 80% of procedures remain open in the United States, may reflect technical complexity, specifically intricate pancreatic, biliary, and gastrointestinal reconstructions and associated risks of severe complications such as postoperative pancreatic fistula [22, 23].

### Intra- and inter-specialty spillover effects and system replacement

While acknowledging that nationwide adoption and institutional familiarity with RAS rapidly expanded in Japan following the major NHI reimbursement revision in 2018 [2, 24], our findings show a pattern consistent with “intra-specialty spillover,” where the presence of a preceding procedure within the same clinical specialty was associated with higher initial adoption of subsequent procedures. This pattern is consistent with the possibility that the diffusion of RAS in Japan may progress through “technology ecosystems” or platform-level integration [7].

Specifically, inter-specialty spillover is hypothesized to reduce organizational costs across an institution. The operational protocols established by pioneering departments such as urology, which include robot setup procedures, the proficiency of operating room nursing and anesthesia staff, institutional credentialing, and administrative billing systems, are reported to serve as an established operational foundation for the entire hospital [12]. Consequently, when subsequent specialties such as gastrointestinal surgery introduce RAS, the temporal and organizational friction of building infrastructure from scratch may be reduced [9]. Furthermore, intra-specialty spillover may be associated with faster technical and psychological synchronization within a shared expert community [18]. The “observability” and “skill reproduction” demonstrated in preceding procedures likely serve as motivators, helping shift surgeons’ preferences from conventional modalities toward robotics [25].

These intra- and inter-specialty spillover dynamics support consideration of a transition from a fragmented, localized evaluation of individual procedures to a holistic, hospital-level system-replacement strategy. To amortize substantial fixed costs, hospitals may benefit from moving beyond single-department use toward maximizing utilization density through a multidisciplinary shared-infrastructure model [4, 20]. Ultimately, institutional efficiency may depend not on whether a single procedure is profitable on its own, but on how systematically the institution replaces its conventional surgical infrastructure with an integrated, multi-specialty robotic platform.

### Theoretical and practical implications

These findings suggest that while the diffusion of RAS in Japan aligns with Rogers’ diffusion theory, it may be associated with the density and composition of expert communities as well as hospital-level economic viability. The rapid technical replacement observed in low-volume procedures is consistent with swift information transmission within highly homophilous, compact networks [4, 26]. Conversely, when applying the concept of the “chasm,” among procedures with a larger annual volume of ≥ 6,000 cases, we identified procedures that either failed to cross the 16.0% threshold or required up to 6 years to surpass it. We hypothesize that this transition zone for crossing the chasm corresponds to the phase in which RAS diffusion expands from specialized centers to general hospitals. Because hospital-level economic viability (estimated at 150–300 procedures per year at a single institution) and the maldistribution of certified proctors may act as major socioeconomic rate-limiting factors hindering adoption, institutional profitability and physician education are suggested to be critical determinants for traversing this chasm [4, 12, 13]. Furthermore, beyond these broad system-level socioeconomic barriers, procedure-specific clinical constraints and structural factors unique to individual specialties also interact to influence the extent of RAS diffusion.

Given these structural characteristics of the diffusion process, a practical implication is that further promotion of RAS may warrant a shift from a narrow focus on procedure-specific superiority toward holistic strategies that optimize the utilization density of existing infrastructure. Within this context, recognizing these two diffusion patterns depending on annual procedural volume, namely a “rapid replacement model” in markets with < 6,000 annual cases and a “model prone to chasm-crossing barriers” in those with ≥ 6,000 cases, may inform strategies for transitioning RAS from localized clinical implementation to system-wide implementation.

### Limitations and future research

This study has four primary limitations. First, while our analysis utilized nationwide administrative claims data, our discussion of spillover effects infers hospital- and practitioner-level mechanisms (such as shared robotic platforms, operational protocols, and accumulated expertise). Due to data constraints, we could not directly link these macro-level national trends to micro-level organizational processes. Second, NDB Open Data lacks facility-level and patient-specific outcomes, which prevented us from directly evaluating relative advantage through clinical superiority. Third, the concealment of data for counts of fewer than 10 cases may underestimate the early dynamics of innovators in small facilities [14, 18]. Fourth, the results are highly dependent on Japan’s universal health insurance and fee schedule, and cannot be directly generalized to healthcare systems in other countries that rely on mixed billing or private insurance.

A key task for future research is to use data from secondary medical service areas (SMSAs), which serve as Japan’s basic regional units for healthcare planning and inpatient care, to investigate regional disparities in proctor distribution and case volumes associated with adoption stalling or delayed traversal of the 16.0% chasm threshold among procedures with an annual volume of ≥ 6,000 cases [15]. Additionally, incorporating analyses in other fields, such as catheter-based cardiovascular interventions, would help generalize the volume-dependent “diffusion paradox” and volume-independent “spillover effect” observed in this study.

## Conclusion

Using comprehensive nationwide claims data, this study analyzed RAS diffusion across clinical specialties in Japan, revealing a volume-dependent diffusion pattern associated with annual surgical volume. Low-volume procedures with an annual surgical volume of < 6,000 cases, concentrated within compact expert communities, showed rapid adoption. Conversely, among procedures with an annual surgical volume of ≥ 6,000 cases, we identified procedures that either failed to cross the 16.0% chasm threshold derived from Rogers’ diffusion theory or required 6 years to surpass it, a pattern consistent with challenges in the shift from adoption among early adopters within specialized centers to uptake among the early majority in general hospitals.

The study also observed an intra-specialty spillover pattern, wherein procedures introduced later within the same specialty achieved higher initial adoption. This pattern may reflect pre-existing infrastructure, trained operating-room teams, and established institutional workflows during the introduction of subsequent procedures within the same clinical specialty.

## Supplementary Information

Below is the link to the electronic supplementary material.

Supplementary Material 1

## Data Availability

The datasets analyzed during this study are public-domain data, which are freely and publicly available from the National Database of Health Insurance Claims and Specific Health Checkups of Japan (NDB) Open Data published by the Ministry of Health, Labour and Welfare of Japan (available at: https://www.mhlw.go.jp/stf/seisakunitsuite/bunya/0000177182.html).
